# Characterization of IgG1 Fc Deamidation at Asparagine 325 and Its Impact on Antibody-dependent Cell-mediated Cytotoxicity and FcγRIIIa Binding

**DOI:** 10.1038/s41598-019-57184-2

**Published:** 2020-01-15

**Authors:** Xiaojun Lu, Lee Ann Machiesky, Niluka De Mel, Qun Du, Weichen Xu, Michael Washabaugh, Xu-Rong Jiang, Jihong Wang

**Affiliations:** 1 Analytical Sciences, BioPharmaceutical Development, R&D, AstraZeneca, One MedImmune Way, Gaithersburg, MD 20878 USA; 2 Department of Antibody Discovery and Protein Engineering, R&D, AstraZeneca, One MedImmune Way, Gaithersburg, MD 20878 USA; 3 Development Quality Biologics and BioVentures, AstraZeneca, One MedImmune Way, Gaithersburg, MD 20878 USA; 4 Present Address: Adimab LLC, 7 Lucent Drive, Lebanon, NH 03766 USA; 5 Present Address: Immunova Therapeutics, Inc., 7707 Fannin Street, Suite 200, Houston, TX 77054 USA; 6 Present Address: Analytical Sciences, Viela Bio, One MedImmune Way, Gaithersburg, MD 20878 USA

**Keywords:** Antibody therapy, Mass spectrometry, Biophysical chemistry

## Abstract

Antibody-dependent cell-mediated cytotoxicity (ADCC) is an important mechanism of action for many therapeutic antibodies. A therapeutic immunoglobulin (Ig) G_1_ monoclonal antibody lost more than half of its ADCC activity after heat stress at 40 °C for 4 months. Size-exclusion and ion-exchange chromatography were used to fractionate various size and charge variants from the stressed IgG_1_. Physicochemical characterization of these fractions revealed that a rarely seen crystallizable fragment (Fc) modification, N325 deamidation, exhibited a positive correlation with the loss of ADCC activity. A further surface plasmon resonance study showed that this modification disrupted the binding between the IgG_1_ Fc and Fcγ receptor IIIa, resulting in decreased ADCC activity of the IgG_1_ antibody. Mutants of N325/D and N325/Q were made to confirm the effect of N325 deamidation on ADCC. We hypothesize that N325 deamidation altered the local three-dimensional structure, which might interfere with the binding and interaction with the effector cell. Because of its impact on biological activity, N325 deamidation is a critical quality attribute for products whose mechanism of action includes ADCC. A thorough understanding of the criticality of N325 deamidation and appropriate monitoring can help ensure the safety and efficacy of IgG_1_ or Fc-fusion products.

## Introduction

Monoclonal antibodies (mAbs) are increasingly used for treatment in organ transplantation and a variety of therapeutic areas, including cancer, autoimmune disorders, and infectious diseases^1^. During the past three decades, the U.S. Food and Drug Administration has approved more than 70 full-length mAbs and related fragments for use in patients, and over 50 more are currently in late-stage clinical development^2,3^. These mAbs, also known as immunoglobulins (Ig), can be divided into five isotypes: IgG, IgM, IgA, IgE, and IgD, each of which has several subtypes. IgG_1_ is the most abundant IgG subtype in human serum^4^, and most current mAb drugs are of this subtype. IgG antibodies consist of two light chains and two heavy chains (HCs) that form three independent regions: two antigen-binding fragments (Fab) and one crystallizable fragment (Fc). The Fab region is responsible for strong binding of the antibody to the target through complementarity-determining regions (CDRs), and the Fc region can interact with different cell surface receptors to mediate various effector functions^5^.

Therapeutic mAbs can either bind directly to specific antigens to disrupt signaling pathways or engage the immune system via different effector functions^5^. Among these effector functions, antibody-dependent cell-mediated cytotoxicity (ADCC) plays a significant role in the *in-vivo* efficacy of many mAbs such as rituximab, trastuzumab and alemtuzumab^4^. Typically, ADCC occurs after the antigen binding region of the antibody binds to the target while the Fc region of the antibody recognizes the Fc γ receptor (FcγR) IIIa expressed on natural killer (NK) cells. The activated NK cells then release cytotoxic granules (perforin and granzymes), leading to apoptosis of the target cell^6^. The importance of ADCC for therapeutic mAbs has been demonstrated by preclinical and clinical studies^7–9^.

Although most human FcγRs, including FcγRIIIa, only have low to medium binding affinity for the Fc region^10^, the binding interface plays a key role in the induction of ADCC by therapeutic mAbs. Amino acid and posttranslational modification (PTM) changes in the interface substantially affect the ability of mAbs to induce ADCC by disrupting or strengthening this binding^11,12^. For example, *N*-linked oligosaccharides at asparagine 297 of IgG Fc regions are essential for ADCC function^13–15^. Removal of Fc N-glycan abolishes ADCC completely. Removal of the fucose from the first *N*-acetylglucosamine of the *N*-linked oligosaccharide at this residue can substantially strengthen the binding affinity of engineered antibodies to FcγRIII and enhance ADCC *in vitro* and *in vivo*^16–19^. To date, reports are limited on how chemical degradations in the Fc region of mAbs could affect ADCC function. Only methionine oxidation has been found to substantially affect the structure and stability of the human IgG_1_ Fc region, but this change has no effect on binding between IgG_1_ and FcγRs^20,21^.

During the product development life cycle, the critical quality attributes (CQAs) of a therapeutic mAb are defined and investigated. A CQA is defined in International Council for Harmonization guideline Q8R2 as “a physical, chemical, biological, or microbiological property or characteristic that should be within an appropriate limit, range, or distribution to ensure the desired product quality.”

The aim of this study was to identify the cause of the biological activity loss after heat stress and understand the degradation pathway of the IgG_1_ antibody. The heat-stressed material was first fractionated by size-exclusion chromatography (SEC) and ion-exchange chromatography (IEC) to separate different size and charge variants. The subsequent physicochemical characterization of these fractions identified N325 deamidation as the major cause of the reduced ADCC activity, and the possible mechanism is also discussed. Finally, we explore the clinical relevance of N325 deamidation, a control strategy to measure N325 deamidation, and correlation of N325 deamidation with stability study data using orthogonal methods.

## Results and Discussion

**Figure 1:**
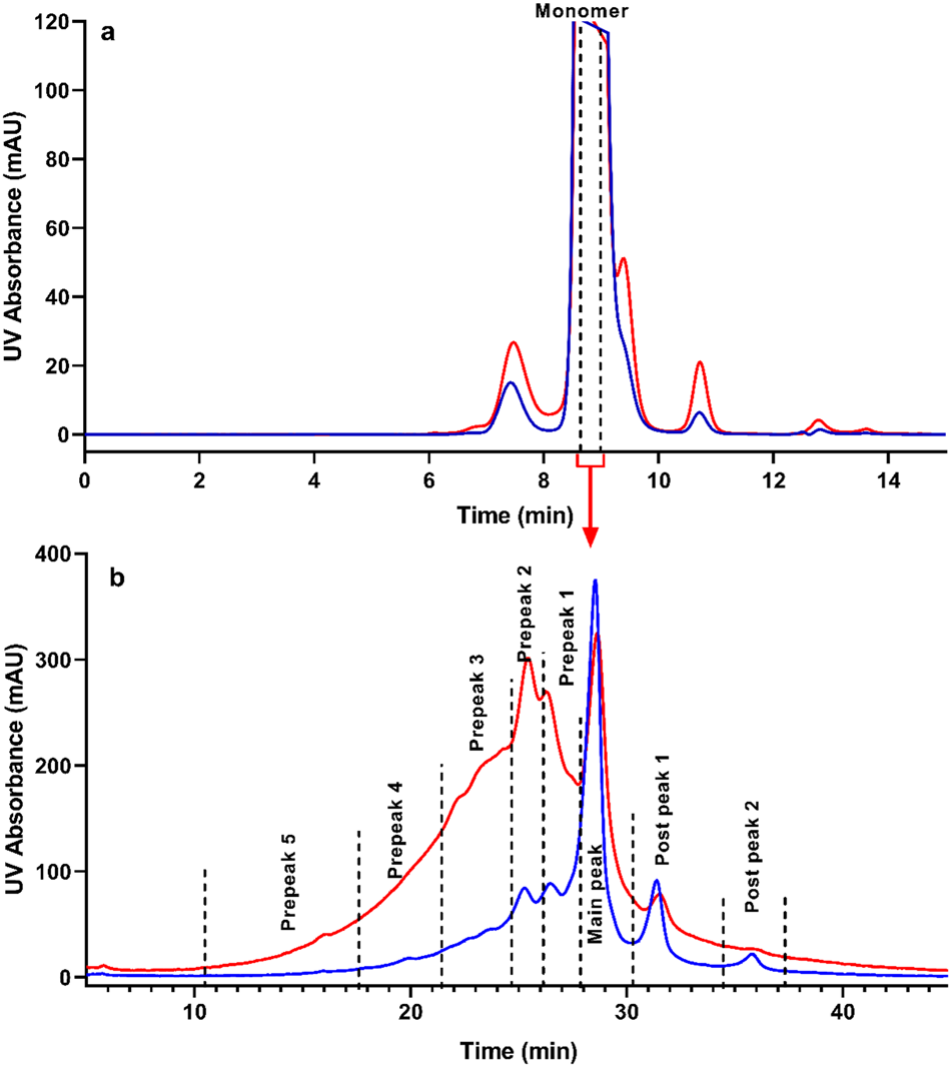
Fractionation of the stressed immunoglobulin G_1_ antibody (**a**) SEC profile of the IgG_1_-A thermally incubated at 25 °C for 10 months. (blue) and at 40 °C for 4 months (red). (**b**) IEC profile of the IgG_1_-A SEC at 25 °C (blue) and 40 °C (red) monomer fractions. The SEC monomer fractions shown within the bracket in Panel a were used for the IEC fractionation shown in Panel b. *mAU* milli-absorbance unit, *UV* ultraviolet.

**Figure 2:**
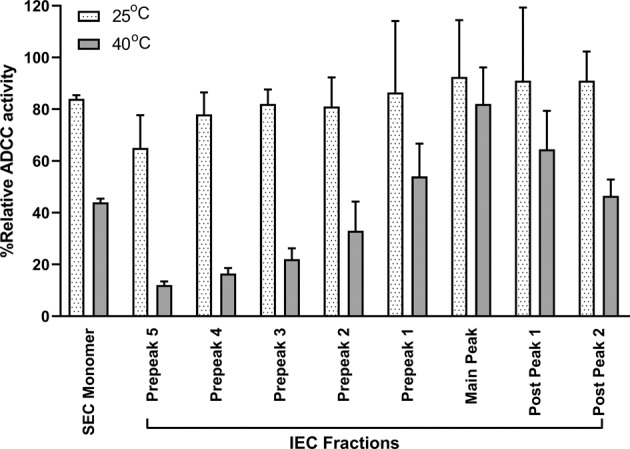
ADCC activity of SEC monomers and IEC fractions. Grey: 25 °C stored samples; black: 40 °C stressed samples.

To understand the degradation pathway and establish a list of CQAs, a therapeutic mAb (IgG_1_-A) with ADCC activity as one of the mechanisms of action was exposed to elevated temperature conditions at 25 °C for 10 months and to stressed conditions at 40 °C for 4 months. Interestingly, although the 25 °C incubated sample was able to retain 94% of ADCC activity, the 40 °C stressed sample exhibited only 35% of ADCC activity. To identify the degradation pathways and determine the cause of the activity loss, SEC was used to separate and quantify the size variants in both heat-treated samples. As observed in the overlay of the two SEC profiles (Fig. 1, Panel a), the 40 °C stressed sample contained slightly more dimers and fragments than the 25 °C incubated sample, but both samples were still predominantly monomeric. Furthermore, the SEC fractions solely enriched with monomers from the 40 °C stressed sample still exhibited less than 50% of ADCC activity (Fig. 2), suggesting that the removal of other size variants, such as dimers and fragments, could not restore the ADCC activity of the antibody.

To further investigate the root cause of ADCC activity loss, IEC was used to separate and fractionate different charge variants in two SEC monomer fractions collected from the 25 °C and 40 °C stressed samples. As shown in Fig. 1, Panel b, IEC fractionation produced five acidic fractions (Prepeak 5 to Prepeak 1), one main fraction (main peak), and two basic fractions (Postpeaks 1 and 2) from each SEC monomer sample. The relative activity of all IEC fractions is shown in Fig. 2. Interestingly, all 40 °C acidic fractions had lower ADCC activity than the 40 °C main peak fraction, whereas most 25 °C acidic fractions exhibited similar activity to their main peak fraction. In addition, the ADCC activity of the 40 °C fractions gradually decreased from main peak (82%) to Prepeak 5 (12%), indicating that these fractions might contain gradually increasing concentrations of certain charge variant(s) that interfere with ADCC activity.

Peptide mapping analysis was used to characterize the IEC fractions to quantify PTMs. Table 1 summarizes the PTMs present at significant levels in the all IgG_1_-A IEC fractions. The results showed that 25 °C main peak, Postpeak 1, and Postpeak 2 contained 6.7%, 48.2% and 73.1% HC C-terminal lysine, respectively. Because each mAb molecule has two identical HCs, these findings confirmed that 25 °C main peak, Postpeak 1, and Postpeak 2 were mainly 0-lysine, 1-lysine and 2-lysine variants, respectively. The fact that all these three fractions showed similar ADCC activity suggests that C-terminal lysine heterogeneity had no effect on the activity. The other four high level PTMs present in acidic fractions were M252 oxidation, N325 deamidation, N384/N389 deamidation, and HC N-terminal pyroglutamate (pE). M252 oxidation was not related to ADCC activity according to Fc receptor binding studies^20^. Although there is no apparent increase from main peak to Prepeak 5 to in 25 °C IEC fractions, the levels of the other three PTMs increased from main peak to Prepeak 5 in 40 °C IEC fractions.

**Table 1:** PTM percentages of the antibody fractions determined by peptide mapping assay.

PTM (%)	Oxidation	Deamidation		HC N-terminal pE	HC C-terminal lysine^b^
Location	M252	N325	N384/N389^a^		
25 °C SEC monomer	13.6	6.5	9.4	3.0	13.9
25 °C IEC Prepeak 5	19.1	9.5	13.0	3.6	5.3
25 °C IEC Prepeak 4	19.3	8.4	12.6	3.7	5.9
25 °C IEC Prepeak 3	18.2	8.3	14.8	3.8	5.9
25 °C IEC Prepeak 2	18.8	10.0	11.6	3.7	6.1
25 °C IEC Prepeak 1	18.3	7.1	17.9	5.8	7.1
25 °C IEC main peak	13.0	3.0	6.6	1.6	6.7
25 °C IEC Postpeak 1	18.4	2.7	6.6	1.4	48.2
25 °C IEC Postpeak 2	22.1	2.3	6.5	0	73.1
40 °C SEC monomer	29.2	29.1	15.4	9.3	7.7
40 °C IEC Prepeak 5	38.5	48.1	22.1	12.1	3.4
40 °C IEC Prepeak 4	38.6	44.5	20.3	12.4	6.3
40 °C IEC Prepeak 3	34.7	40.6	18.3	10.4	4.4
40 °C IEC Prepeak 2	36.8	31.4	12.6	7.6	4.6
40 °C IEC Prepeak 1	34.8	22.6	16.3	9.4	6.5
40 °C IEC main peak	38.3	9.2	9.2	4.3	10.8
40 °C IEC Postpeak 1	38.6	12.7	10.9	5.1	31.9
40 °C IEC Postpeak 2	38.5	14.3	10.9	7.0	35.9

All amino acid positions are numbered according to the EU index. PTM posttranslational modification, HC heavy chain, pE pyroglutamate, SEC size-exclusion chromatography, IEC ion exchange chromatography

^a^N384 and N389 are located in the same tryptic peptide, so their deamidation levels were calculated together.

^b^To maximize accuracy, the ultraviolet signals of Lys-C peptide covering the HC C-terminus were used to calculate lysine heterogeneity.

Because the N-terminus is not proximal to the CDR, N-terminal heterogeneity is unlikely to interfere with antigen binding. It is reported that N-terminal pE does not affect the biological activity of a mAb that relies on antigen binding^22^. Furthermore, we stressed the lyophilized IgG_1_-A at 40 °C for 3 years and used IEC fractionation to enrich the HC pE variants. The IEC fraction with pE proportion to approximately 50% of molecules exhibited 88% of the ADCC activity of the reference standard which only contained 2% of pE-containing molecules (the results will be summarized and reported in a separate manuscript). Thus, it can be concluded that HC N-terminal pE level is not related to the loss of ADCC activity in IgG_1_-A.

To assess the correlation between N325 or N384/N389 deamidation and ADCC activity, we incubated IgG_1_-A in IgG-depleted human serum at 37 °C for 4 weeks to generate considerable levels of N384/N389 deamidation as reported previously^23^.

Tryptic peptide mapping analysis revealed that after serum incubation N384/N389 deamidation increased significantly (from 4.4% to 35.0%); however, N325 deamidation did not increase (from 2.0% to 2.1%). The relative ADCC activity of the serum-incubated sample was 88%, indicating that N384/N389 deamidation had no impact on ADCC activity.

**Figure 3:**
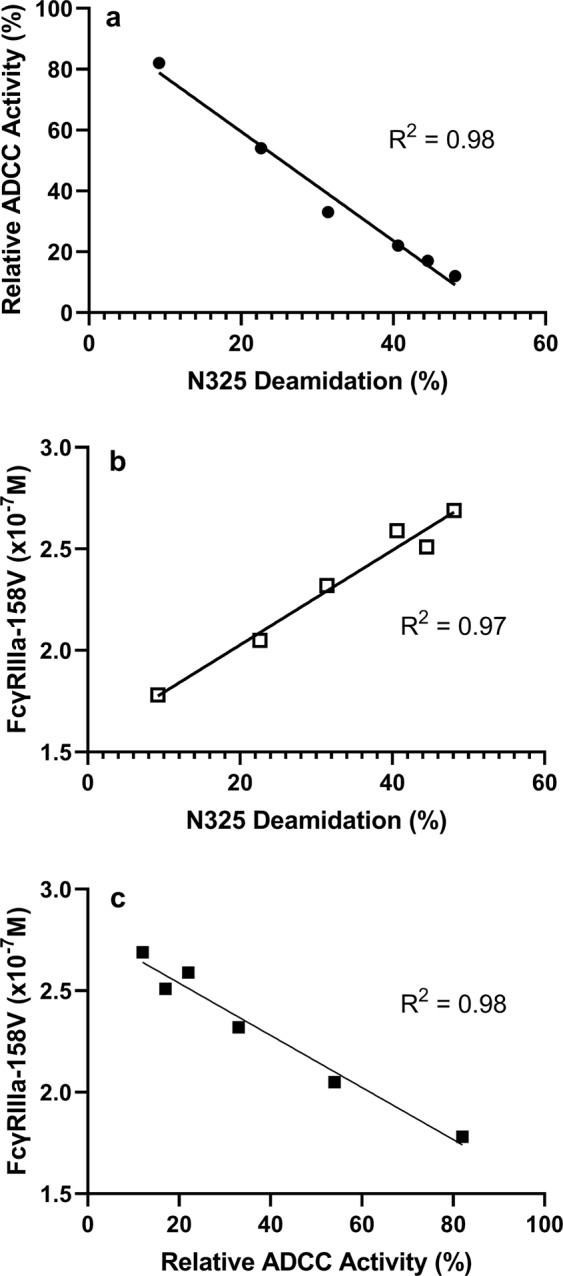
(**a**) ADCC activity vs. N325 deamidation for selected 40 °C ionic exchange chromatography fractions. Linear regression was used to evaluate the relationship between ADCC activity and N325 deamidation. (**b**) FcγRIIIa binding vs. N325 deamidation for selected 40 °C ionic exchange chromatography fractions. Linear regression was used to evaluate the relationship between FcγRIIIa binding and N325 deamidation. (**c**) FcγRIIIa binding vs. ADCC activity for 40 °C IEC prepeak and main peak fractions. Linear regression was used to evaluate the relationship between FcγRIIIa binding and ADCC activity.

Based on these results, we concluded that N325 deamidation is likely to be the primary cause of ADCC activity loss in the 40 °C stressed IEC prepeak fractions. When the relative ADCC activity of the fractions was plotted against N325 deamidation, linear regression confirmed a strong negative correlation between N325 deamidation and ADCC activity (R^2^ = 0.98; Fig. 3, Panel a). These results suggest that disruption of ADCC effector function was predominantly the result of N325 deamidation.

The ADCC effector function requires not only binding to the antigen on target cells through antibody CDRs, but also binding to FcγRIIIa expressed on effector cells (e.g. NK cells) through the Fc region. To better understand how N325 deamidation affects ADCC activity of the antibody, both antigen binding and FcγRIIIa binding of 40 °C IEC fractions were analyzed using SPR methods. The relative antigen and FcγRIIIa binding activities are summarized in Supplementary Information (Supplementary Table S1). For antigen binding, only the 40 °C Prepeak 5 fraction exhibited a reduced relative binding due to increased level of CDR fragmentation. For FcγRIIIa binding, however, binding affinity to FcγRIIIa declined gradually from the IEC main peak to the prepeak5. The FcγRIIIa binding of the 40 °C IEC fractions showed a strongly negative correlation with the N325 deamidation. The linear regression fitting determined an R^2^ value of 0.97 (Fig. 3b). In contrast, no such correlation could be established for antigen binding. Therefore, the SPR results suggested that N325 deamidation had minimum impact on the antigen binding ability of the antibody but did decrease its FcγRIIIa binding, leading to a loss of ADCC activity.

Even though the FcγRIIIa binding only decreased by about 2-fold (40%), the ADCC activity loss was almost 90% when the N325 deamidation level reached 40–45% from the average of the two HCs. The correlation of the changes between FcγRIIIa binding and ADCC were linear with regression of the R^2^ value of 0.98 (Fig. 3, Panel c). The difference in the percentage change between the two assays was most likely due to the assay format. As reported by Chung *et al*.^24^, the binding of FcγRIIIa-158V increased only twofold, but ADCC activity increased threefold when afucosylation levels changed from 0% to 10%, whereas the binding of FcγRIIIa-158F increased by more than threefold in the same condition.

N325 deamidation produced a more profound decrease in ADCC activity assay than the FcγRIIIa binding assay. One hypothesis could be that a small degree of conformational change may affect the engagement of effector cells to target cells through FcγRIIIa binding. This effect can be more effectively measured in a cellular system in which both effector and target cells exist, whereas SPR does not have the capability of measuring conformational changes during cell-to-cell interactions. The difference between the cell-based and SPR binding results is consistent with a previous observation that interactions of human FcγRIII with IgG had different binding kinetics and affinity when using native cell-bound receptor versus the recombinant protein [25]. The membrane anchor property had an impact on FcγRIII binding, which was probably due to conformational differences between the membrane receptor isoforms^25^.

Another potential explanation of the different impact of N325 deamidation on ADCC activity and FcγRIIIa binding observed in our study could involve formation of asymmetrical Fc with one deamidated HC. Crystal structures of novel asymmetrically engineered Fc variants with improved affinity for FcγRs have been reported with amino acid mutations in Fc at one of the HCs^26^. In our study, the deamidation at N325 could occur mainly at one of the HCs to form asymmetrical Fc for enriched deamidation of heat stress material. N325 deamidation reached as high as 48.5% for prepeak 5 when measured by peptide mapping, which can account for up to 97% IgG_1_ with one HC deamidated. FcγRIIIa might still bind to asymmetrical Fc with lower affinity than nondeamidated Fc. However, ADCC, which requires effector cell engagement to target cells, can be eliminated from asymmetrical Fc because of the difference in structure flexibility and orientation of the FcγRIIIa binding.

**Figure 4:**
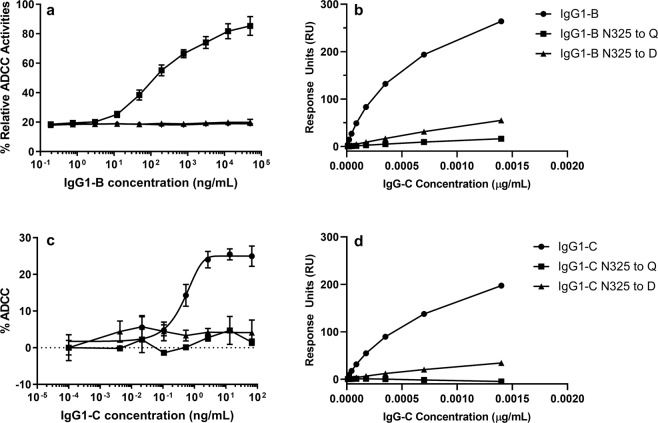
ADCC activity and crystallizable fragment γ receptor IIIa binding for N325D and N325Q mutants. Immunoglobulin (Ig) G1-B and IgG_1_-B N325D/N325Q mutants were tested in (**a**) the ADCC reporter gene assay and in (**b**) the Fcγ receptor binding assay. IgG_1_-C and IgG_1_-C N325D/N325Q mutants were tested in (**c**) the OE21 ADCC assay and (**d**) the Fcγ receptor binding assay. The ADCC activity of IgG_1_-B and IgG_1_-C was eliminated because of the mutation of N325 to N325D/Q, and the loss of ADCC is largely attributable to the loss of binding to Fcγ receptor IIIa.

To further confirm that N325 deamidation alone can eliminate ADCC activity on IgG_1_s, site-specific mutants at N325 to D or Q were generated using two additional IgG_1_ molecules (IgG_1_-B and IgG_1_-C). The mutations were confirmed by mass spectrometry analysis, and the glycosylation was characterized by hydrophilic interaction liquid chromatography to ensure that the parent and mutants had similar afucosylation levels. IgG_1_-B targets an antigen on T cells, and ADCC of IgG_1_-B was measured using a reporter gene assay that detected the effector cell signaling using the parent molecule as a reference. IgG_1_-C targets an antigen on OE21 cells, and ADCC of IgG_1_-C was measured in a flow-based cell cytotoxicity assay using afucosylated anti-epidermal growth factor receptor antibody panitumumab as a reference. Mutations at N325 resulted in both complete loss of ADCC and diminished FcγRIIIa binding (Fig. 4). In addition to the data shown above for the N325/D mutant, the N325/Q mutation showed diminished FcγRIIIa binding and ADCC activity for IgG_1_-B. Similar results were observed for another IgG_1_ antibody targeting epidermal growth factor receptor (IgG_1_-C), and mutations of N325/D and N325/Q decreased both FcγRIIIa binding and ADCC activities. These results indicate the essential role of N325 for FcγRIIIa binding and effector function, which supports our conclusion that N325 deamidation caused ADCC activity loss.

To explain why N325 deamidation affected ADCC activity, we examined several published crystal structures of Fc:FcγRIII complexes^15,16,27^. Sondermann *et al*. reported the structure of a complex formed by soluble FcγRIII and a human IgG_1_ Fc fragment^27^, and Radaev *et al*. crystalized a similar complex with human FcγRIIIb^28^. Both groups reported 1:1 stoichiometric complex with a similar binding interface between Fc and the receptors, and one key element for the interface is a hydrophobic core formed mainly by P329 from Fc with W90 and W113 from the receptor. The FcγRIII used in these two studies were produced in *Escherichia coli* and were not glycosylated, but Ferrara *et al*. later confirmed that the complex formed by glycosylated FcγRIIIa and Fc was closely related to these published structures^16^. As one of the major contact areas in the defined interface, the hydrophobic core surrounding P329 plays a key role in ADCC function, and alanine substitution of this residue abrogates the binding and abolishes ADCC activity^29^. Disturbance of neighboring amino acids (e.g., alanine substitution of K322) also leads to a reduction in FcγRIIIa binding affinity and ADCC function^30^. The fact that N325 is in close proximity to P329 suggests that N325 deamidation could alter the local three-dimensional structure, which might change the binding interface or interfere with the binding interface between Fc and its receptors, resulting in the loss of ADCC activity.

As a major degradation pathway for therapeutic mAbs, asparagine deamidation has been widely studied and well documented^31–39^. Unlike many other identified deamidation sites, N325 has not been the focus of many degradation studies because it is not associated with regular motifs that could be prone to deamidation, such as Asn-Gly, Asn-Ser, Asn-Thr, and Asn-Asn. It was not until 2006 that Liu *et al*. first reported deamidation at N325 when they incubated a human IgG_1_ antibody at 40 °C and pH 5.2 for 6 months; however, the identification was based only on the isotopic distribution change caused by co-eluting intact and deamidated peptides^34^. Chung *et al*. have also recently observed this modification in the company’s IgG_1_ mAbs^38^ because of the well-conserved amino acid sequence of human IgG Fc regions. Pace *et al*. found that the pH dependence of the Fc deamidation rate was lowest at pH 6.3 and had upswings at both lower and higher pH levels. The increased deamidation rates observed at pH 6.3 were ascribed to three sites, N315, N384, and N389, and the greater rate at pH~5.5 was tentatively assigned to N325^35^. Subsequently, Zhang *et al*. used chymotrypsin peptide mapping to fully characterize N325 deamidation and confirmed that at mildly acidic pH, Fc deamidation occurred mainly at this site^38^. Based on the comparison of different IgG_1_ Fc structures in the Protein Data Bank that were crystalized at different pH (from 4.0 to 7.0), Yan *et al*. revealed a negative correlation between the solvent accessibility for N325 and pH even when the site is considered to be buried in the local three-dimensional structure at a neutral pH^40^. N325 is exposed only at acidic pH and subsequently is deamidated at high temperatures, providing an explanation for why N325 deamidation occurs only in formulation buffer and under heat stress conditions.

**Figure 5:**
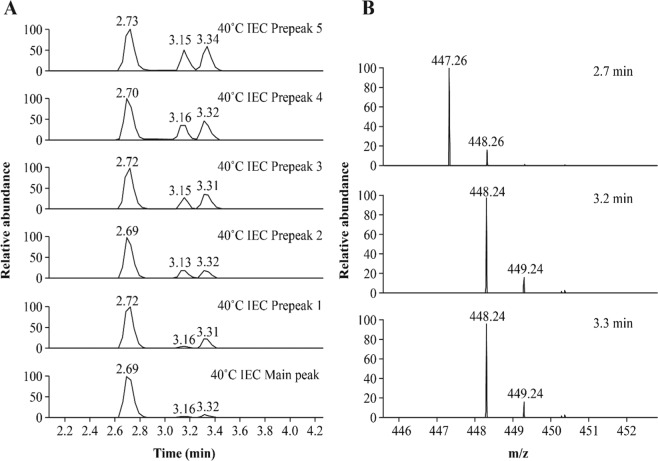
Tryptic peptide mapping results of the N325-containing VSNK peptide for 40 °C ion exchange chromatography prepeaks and main peak fractions. (**A**) Extracted ion chromatography of the VSNK and deamidated VSNK peptides. The fraction name is indicated on the right of each panel. (**B**) Mass spectrometry spectra corresponding to 2.7 minutes (top), 3.2 minutes (middle), and 3.3 minutes (bottom).

Site N325 has been overlooked in the past partially because of the technical challenge of tryptic peptide mapping, the standard methodology used to characterize the primary sequence of a protein and any possible PTMs. Tryptic digestion of IgG_1_ antibodies yields a tetrapeptide 323-Val-Ser-Asn-Lys (VSNK)-326, a hydrophilic peptide that is often eluted near the void volume of reversed-phase high-performance liquid chromatography. In previous literature, this peptide was co-eluted with its deamidated product, which led to an increased second isotopic peak within its isotopic distribution^34^. As shown in Fig. 5, our tryptic peptide mapping data revealed that a single-charged ion at mass/charge ratio (m/z) of 447.26 was eluted at 2.7 minutes in the separation conditions described above, which matches the theoretical molecular weight of the VSNK peptide. Surprisingly, two single-charged ions also at m/z 448.24 were also eluted separately at 3.2 minutes and 3.3 minutes, matching the molecular weight of the deamidated VSNK peptide. To further confirm the deamidation site, conventional collision-induced dissociation tandem mass spectra of three peptides were carefully examined. The MS/MS spectra are shown in Supplrmentary Fig. 1S, which clearly identified the three peptides as one VSNK and two deamidated VSNK peptides.

**Figure 6:**
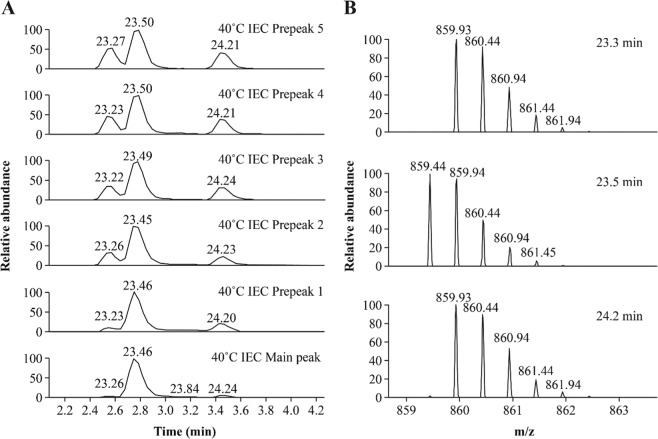
Glu-C peptide mapping results of the N325-containing peptide YKCKVSNKALPAPIE for 40 °C IEC prepeak and main peak fractions. The fraction name is provided on the right side of each panel. (**A**) Extracted ion chromatography of YKCKVSNKALPAPIE and its deamidated peptide. (**B**) Mass spectrometry spectra corresponding to 23.3 minutes (top), 23.5 minutes (middle), and 24.2 minutes (bottom). *m/z* mass/charge ratio.

To further confirm the identification and quantification of the N325 deamidation, we replaced trypsin with Glu-C to repeat peptide mapping analysis with selected 40 °C IEC fractions. As shown in Fig. 6, a 15-residue peptide, 319-YKCKVSNKALPAPIE-333, was eluted at 23.5 minutes, as were two deamidated peptides at 23.2 minutes and 24.2 minutes. Similar to tryptic digested peptide mapping, the identities of the peptides were confirmed by subsequent tandem mass spectra, which showed that all b and y ions containing N325 from peaks at 23.2 minutes and 24.2 minutes had a 1 Da increment compared with those at 23.5 minutes (Supplementary Fig. 2S). Based on the MS data from Glu-C peptide mapping, the deamidation levels of N325 were calculated to be 47.5% for Prepeak 5, 42.0% for Prepeak 4, 38.3% for Prepeak 3, 32.0% for Prepeak 2, 21.5% for Prepeak 1, and 9.0% for the main peak. These findings are consistent with the results from tryptic peptide mapping, suggesting that our tryptic peptide mapping assay could detect the N325 deamidated products and accurately quantify the degree of PTM.

## Conclusion

Because the amino acid sequence of the Fc region is well conserved among all human IgGs, any PTMs observed in this region could potentially affect many therapeutic antibodies. In our study, a rarely observed modification, N325 deamidation, abolished the ADCC activity of an IgG_1_ antibody. Mutations of N325/D and N325/Q decreased both FcγRIIIa binding and ADCC activities. Our data indicate that the abrogated ADCC activity due to N325 deamidation was mainly caused by disruption at the binding interface of the Fc and FcγRIIIa since no correlation was found between target binding and N325 deamidation. As discussed by Yan *et al*., Fc structures could present in different conformations in acidic or neutral conditions^40^. We conclude that N325 can become exposed in acidic conditions and deamidated at elevated temperatures. Once N325 was deamidated, it altered the conformation that caused the decrease in FcγRIIIa binding and ADCC activity.

Because N325 deamidation occurs mainly in low pH and high temperature stress conditions, it is unlikely to occur during a controlled manufacturing process or in appropriately controlled storage and shipping conditions. However, for Class I therapeutic antibodies or Fc fusion proteins in which ADCC effector function is part of the mechanism of action, N325 deamidation is considered a CQA. Therefore, an appropriate risk assessment and control strategy should be in place to ensure product safety and efficacy. In addition, mutation to the N325 site, either alone or in combination with glycoengineering, could be useful to reduce ADCC for molecules when ADCC is not desired.

## Methods

IgG_1_ samples were stressed and fractionated by SEC and IEC. The fractions were tested for degration products by peptide mapping assay, and then correlated with their ADCC activities, antigen binding, and FcγRIIIa binding. Mutants IgG_1_s were made to confirm the finding.

### Sample degradation

The recombinant antibody (IgG_1_-A) used in this study was expressed in Chinese hamster ovary cells and purified using the typical downstream process at AstraZeneca (Gaithersburg, MD) and formulated in histidine buffer at pH 6.0 and a concentration of 100 mg/mL. For the degradation study, IgG_1_-A was exposed to two elevated-temperature conditions: 25 °C for 10 months and 40 °C for 4 months.

For the serum incubation study, normal human serum from Bioreclamation (Hicksville, NY) was filtered through 0.22 µm filters and passed over a Protein A Sepharose Fast Flow column (GE Healthcare Life Sciences, Pittsburgh, PA; 1.15 cm × 12.1 cm) twice for IgG depletion. IgG_1_-A was spiked into the IgG-depleted serum to achieve a final concentration of 1 mg/mL and incubated at 37 °C for 4 weeks. The sample was then purified by protein A chromatography, neutralized, and buffer exchanged into 20 mM sodium phosphate at pH 7.0 and concentrated to approximately 4 mg/mL before testing.

### Size exclusion chromatography (SEC) fractionation

Each stressed sample was injected onto a TSK-gel G3000SWXL column (Tosoh Bioscience, King of Prussia, PA; 7.8 mm × 30 cm) at ambient temperature. The sample was eluted isocratically with a mobile phase composed of 0.1 M sodium phosphate and 0.1 M sodium sulfate at pH 6.8 and a flow rate of 1.0 mL/min. Eluted protein was detected using ultraviolet (UV) absorbance at 280 nm, and fractions were collected using a fraction collector. All fractions from the center of the SEC monomer peak were pooled and concentrated for each stressed sample (Fig. 1, Panel a).

### Ion-exchange chromatography (IEC) fractionation

SEC monomer fractions for each stressed sample were injected onto a ProPac WCX-10 semipreparative column (Thermo Fisher Scientific, Waltham, MA; 9 mm × 250 mm) at ambient temperature. The samples were eluted in a salt gradient from 25–65% mobile phase B for 40 minutes with mobile phase A (composed of 20 mM sodium phosphate at pH 7.0) and mobile phase B (composed of 20 mM sodium phosphate and 100 mM sodium chloride at pH 7.0) at a flow rate of 1.5 mL/min. The eluted protein was detected using UV absorbance at 220 nm, and fractions were collected using a fraction collector. The fractions from several injections were pooled and concentrated. As shown in Fig. 1, Panel b, eight IEC fractions were collected for each stressed sample.

### Mutant preparation

Single mutations (N325D and N325Q) were introduced into the Fc region using polymerase chain reaction overlap extension with mutagenic primers. DNA encoding the mutated Fc regions was cloned into an IgG_1_ expression vector (AstraZeneca, Gaithersburg, MD) through restriction enzyme digestion and ligation-based molecular cloning. The expression vector was transfected transiently into G22 Chinese hamster ovary cells using polyethylenimine (AstraZeneca) according to the manufacturer’s instructions. After transfection, the medium was cultured for 10 days, filtered through a 0.22 μm sterile filter, and purified using the standard mAb purification procedure at AstraZeneca.

### ADCC assay

For IgG_1_-A and IgG_1_-B, the ADCC reporter gene assay used both target cells (CTLL2, a murine cytotoxic T-cell line stably expressing the target antigen) and effector cells (NK92 human NK cell line engineered to express FcγRIIIa and a luciferase reporter gene driven by the nuclear factor of activated T cells [NFAT] promoter). The effector cell (NK92/NFAT) binds to the Fc region of the bound antibody via FcγRIIIa, inducing ADCC of the target cell. The ADCC activity is strongly correlated with the activation of the NFAT transcription factor, which also activates the luciferase gene.

The amount of luciferase generated was measured using the Steady-Glo Luciferase Assay System (Promega Corporation, Madison, WI). The luminescence produced was proportional to the ADCC activity and was quantified by an EnVision Multilabel Plate Reader (Perkin Elmer, Waltham, MA). We then determined the relative ADCC activity of the test sample by dividing the half-maximal effective concentration of the reference standard by that of the test sample and then multiplying the quotient by 100. ADCC activities for IgG-C were measured by a flow-based cell enumeration method using the CellTrace™ CFSE Cell Proliferation Kit (Thermo Fisher Scientific).

For IgG_1_-C, OE21 target cells were seeded in 96-well plates at a density of 2 × 10^4^ cells per well in RPMI-1640 medium without phenol red, and the medium was supplemented with 5% fetal bovine serum. A human NK cell line (KC1333) from a malignant non-Hodgkin lymphoma transgenic for human CD16 (FcgRIIIA) and FceRIg (Xcellerex/Biowa, Marlborough, MA) was mixed with target cells at an effector to target cell incubated for 16 hours at 37 °C in a 5% CO_2_ atmosphere. After treatment, the cells were exposed to CellTrace CFSE reagents and analyzed on an LSR II flow cytometer (BD Biosciences, San Jose, CA) using FACSDiva software, and samples were analyzed with FlowJo software.

### Peptide mapping assay

For trypsin and Glu-C digestion, samples were denatured by adding 8 M guanidine, 130 mM Tris, 1 mM ethylenediaminetetraacetic acid (EDTA), pH 7.6 denaturing buffer. The samples were then reduced using dithiothreitol and alkylated using iodoacetamide. The reduced and alkylated samples were buffer exchanged into a solution containing 2 M urea and 100 mM Tris at pH 8.0 using an Amicon spin filter (EMD Millipore, Billerica, MA; molecular weight cut-off of 10 kDa); Trypsin or Glu-C was then added at an enzyme-to-protein ratio of 1:12.5 to the spin filter and incubated at 37 °C for 4 hours. The digested samples were collected from the spin filters, and the digestion was quenched with trifluoroacetic acid.

For Lys-C digestion, samples were first alkylated with N-ethylmaleimide to cap any free thiol. The samples were then denatured in the presence of 7 M guanidine at 37 °C for 30 minutes. The denatured samples were diluted about fourfold with 100 mM phosphate buffer and 0.1 mM EDTA and then digested by endoproteinase Lys-C at an enzyme-to-protein ratio of 1:10. The reaction mixtures were incubated at 37 °C overnight. The same amount of Lys-C was added next morning, followed by incubation at 37 °C for 4–6 hours. The digested samples were stored at −80 °C before analysis.

Peptides produced by enzymatic digestion were eluted on an Acquity Ultra Performance liquid chromatography system (Waters, Milford, MA) equipped with an ethylene bridged hybrid C18 reversed-phase column (1.7 µm, 2.1 × 150 mm) using a gradient of 0–60% acetonitrile at a flow rate of 0.2 mL/min (total elution time of 76 minutes). Peptides separated on the column were identified by a UV detector and analyzed using an LTQ Orbitrap mass spectrometer (Thermo Fisher Scientific). Peak identification was based on both the exact monoisotopic mass and the tandem mass spectrum of the target ion. HC C-terminal lysine heterogeneity was evaluated based on UV signals. The quantitation of all other peptides was based on peak areas from the extracted ion chromatography of corresponding ions.

### Antigen-binding assay

Soluble antigen was immobilized on a carboxymethyl-dextran sensor surface using an automated procedure on the Biacore C surface plasmon resonance (SPR) biosensor (GE Healthcare Life Sciences). Reference standard, assay control, and test samples were prepared at a concentration of 100 µg/mL in the assay running buffer [HBS-EP+; 10 mM 4-(2-hydroxyethyl)-1-piperazineethanesulfonic acid, 150 mM sodium chloride, 3 mM EDTA, and 0.05% polysorbate-20 at pH 7.4] and serially diluted from 100 µg/mL to 0.017 µg/mL. Reference standard and test samples were injected onto the sensor surface for 120 seconds at 40 µL/min. The sensor surface was regenerated with a 15-second injection of 3 M magnesium chloride at 40 µL/min. The percent relative binding of the test sample to the reference standard was determined by dividing the half-maximal effective concentration of the reference standard response curve by that of the test sample response curve and multiplying by 100.

### FcγRIIIa-158V binding assay

An antihistidine tag mAb from AbD Serotec (Kidlington, UK) was immobilized to two flow cells of a CM5 sensor chip using an automated procedure on a Biacore T200 biosensor (GE Healthcare Life Sciences). One flow cell served as a reference surface and the second as the experimental surface. A histidine-tagged FcγRIIIa-158 V receptor was diluted to 10 µg/mL in assay running buffer (HBS-EP+) and injected over the sensor surface at 10 µL/min for 30–40 seconds. Serially diluted protein samples (700, 350, 175, 87.5, 43.8, 21.9, 10.9, and 0 nM) were then injected over the FcγRIIIa-158 V capture surface at a flow rate of 30 µL/min for 60 seconds and allowed to dissociate for 120 seconds. The anti-histidine tag mAb sensor surface was regenerated with 20 mM hydrochloric acid injected at a rate of 30 µL/min for 30 seconds. Data were reference flow cell subtracted and buffer blank subtracted, then fitted to a steady-state affinity model using Biacore T200 evaluation software (version 2.0) to determine the equilibrium dissociation constants (K_D_) of binding. The percent relative binding was calculated by dividing the K_D_ of reference material by the K_D_ of the sample and multiplying by 100.

## Supplementary information

Supplementary material.
