# Supplementary material for: Characterization of IgG1 Fc Deamidation at Asparagine 325 and Its Impact on Antibody-dependent Cell-mediated Cytotoxicity and FcγRIIIa Binding
Source: Sci Rep. 2020 Jan 15;10:383. doi: 10.1038/s41598-019-57184-2 (PMC6962426; doi:10.1038/s41598-019-57184-2)

Supplementary materials

Characterization of IgG1 Fc Deamidation at Asparagine 325 and Its Impact on Antibody-dependent Cell-mediated Cytotoxicity and FcγRIIIa Binding

Xiaojun Lu, LeeAnn Machiesky, Niluka De Mel, Qun Du, Weichen Xu, Michael Washabaugh, Xu-Rong Jiang, Jihong Wang

Supplementary Table S1 Binding of selected 40°C IEC fractions to antigen and FcγRIIIa

Sample description	Relative antigen binding through CDR (%)	Relative FcγRIIIa-158V KD (nM)
40°C IEC Prepeak 5	72	269
40°C IEC Prepeak 4	94	251
40°C IEC Prepeak 3	94	259
40°C IEC Prepeak 2	99	232
40°C IEC Prepeak 1	101	205
40°C IEC main peak	98	178

IEC ionic exchange chromatography, *FcγRIIIa* crystallizable fragment γ receptor IIIa, *CDR* complementarity-determining region

Fig. 1S Tandem mass spectrometry spectra of the VSNK and deamidated VSNK peptides corresponding to peaks from Figure 5, Panel b. Peaks at (a) 2.7 minutes, (b) 3.2 minutes, and (c) 3.3 minutes. The interpretation of these product ion spectra is summarized at the top of the figure. *m/z* mass/charge ratio.

The same y_1 ions were observed from all peptides, and their molecular weights matched the theoretical values exactly. Whereas the y_2 and y_3 ions from the first peptide still had the theoretical molecular weight, both y_2 and y_3 ions from the second and third peptides had increments of 1 Da. This MS/MS spectra clearly identified the three peptides as one VSNK and two deamidated VSNK peptides.

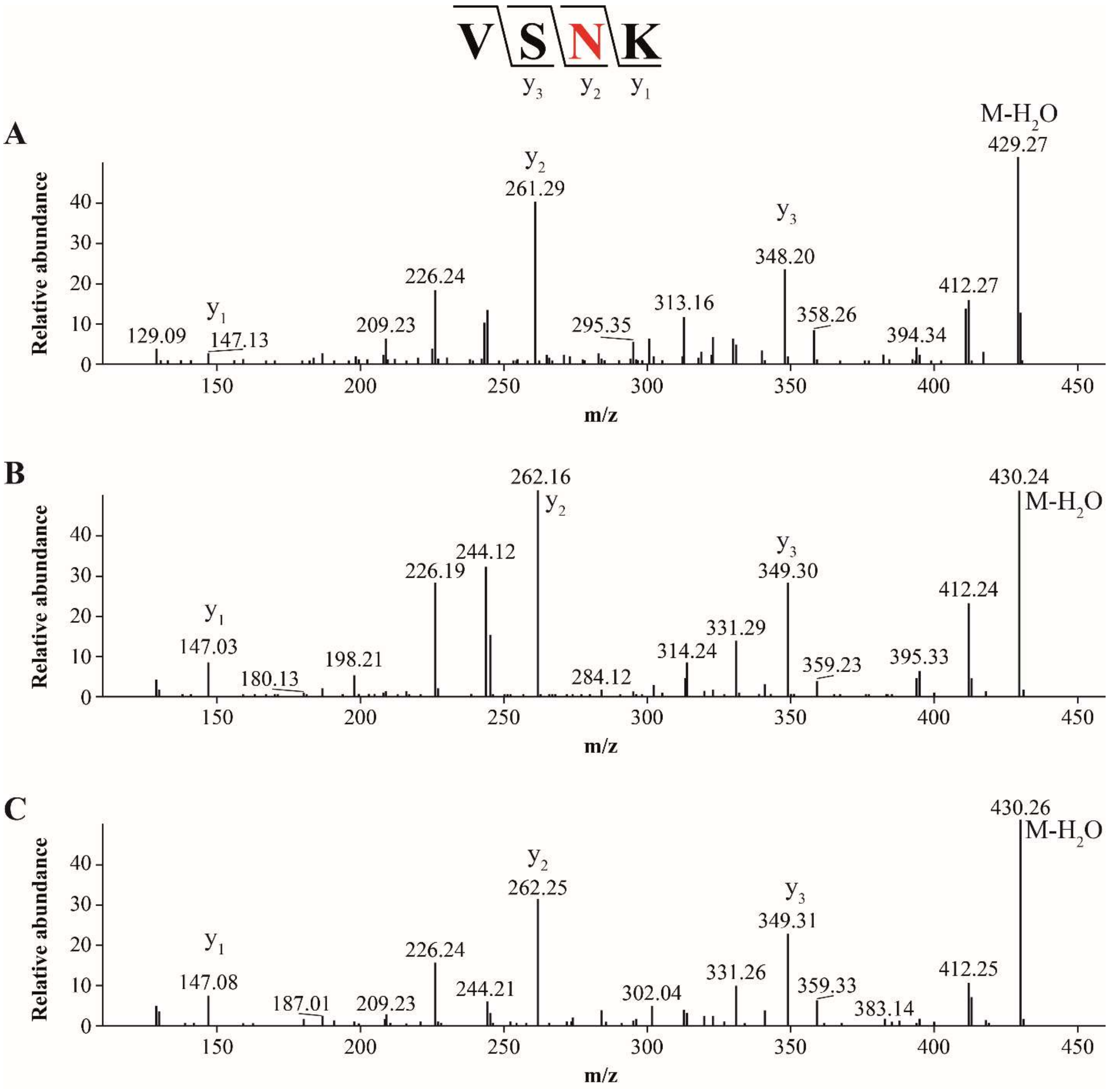

Fig. 2S Tandem mass spectrometry spectra of YKCKVSNKALPAPIE and its deamidated peptides corresponding to peaks from Figure 6, Panel b. Peaks shown were at (a) 23.3 minutes, (b) 23.5 minutes, and (c) 24.2 minutes. The interpretation of these product ion spectra is summarized at the top of the figure. *m/z* mass/charge ratio.

All b and y ions containing N325 from peaks at 23.2 minutes and 24.2 minutes had a 1 Da increment compared with those at 23.5 minutes.

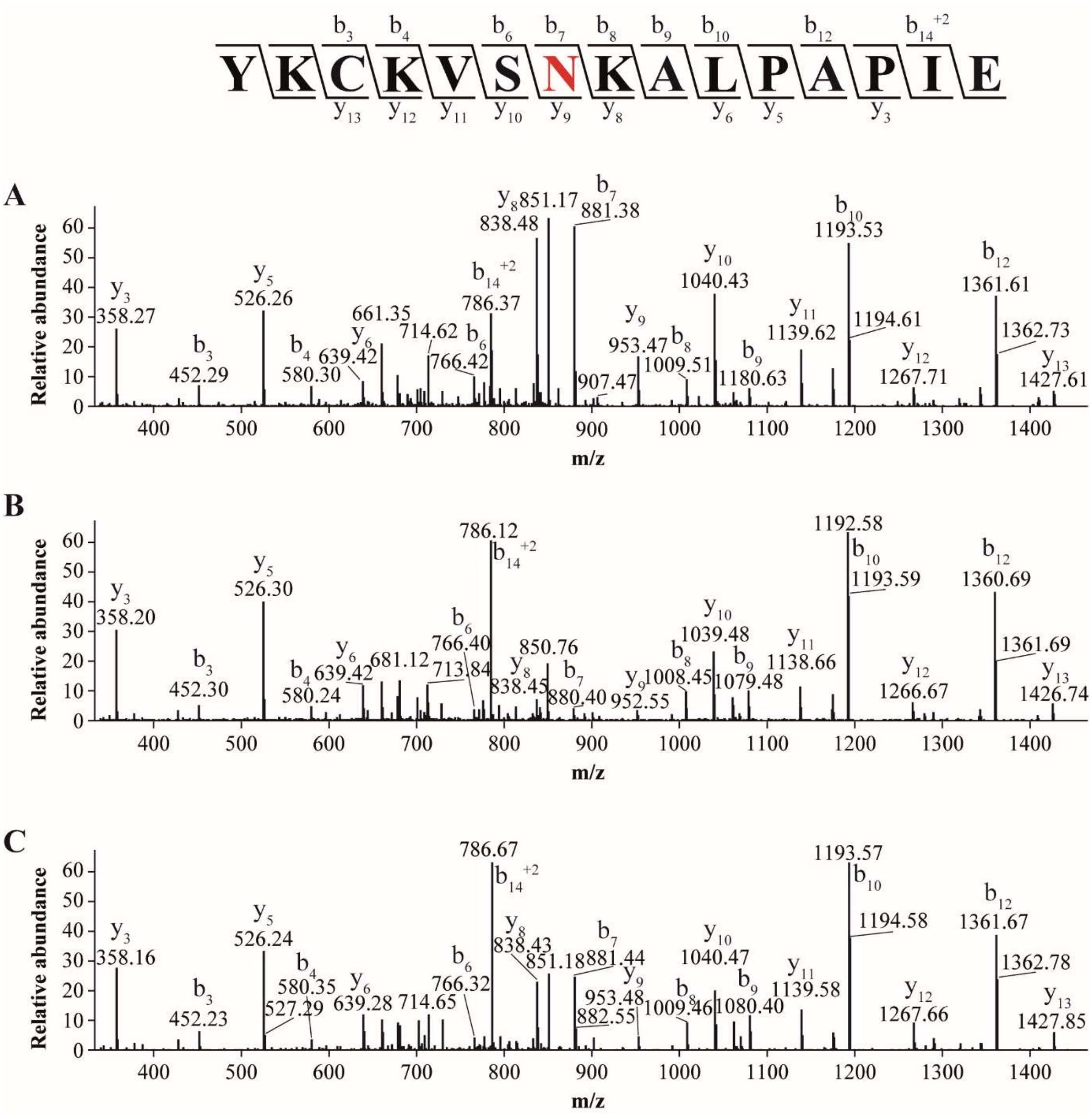

Supplement: Supplementary file 1 — Supplementary material. [file 41598_2019_57184_MOESM1_ESM.pdf]
